# Supplementary material for: Complete Range of the Universal mtDNA Gene Pool and High Genetic Diversity in the Thai Dog Population
Source: Genes (Basel). 2020 Feb 27;11(3):253. doi: 10.3390/genes11030253 (PMC7140826; doi:10.3390/genes11030253)

Figure S1: MS networks showing relationships among haplotypes, including the unique haplotypes in haplogroups A and D from Nigeria not shown in Figure 3. Representation of haplotypes are indicated for Thailand, Nigeria and sub-Saharan Africa excluding Nigeria. Circles represent haplotypes, lines connecting haplotypes represent a single substitution step, and black dots represent hypothetical intermediary haplotypes. The 18 universal haplotypes (UTs) are indicated by black bold circles. The six haplogroups A-F are presented as separate networks. The ten sub-haplogroups a1-a6, b1-b2, c1-c2, are separated by dashed lines. For each geographical region, colored circles indicate haplotypes represented in that region, yellow indicating a haplotype shared with other regions and red indicating haplotypes unique to the region. For clarity of the picture, some connections between Nigerian unique haplotypes in haplogroup A are not shown.

Figure S1a. MS networks showing relationships among haplotypes in Thailand

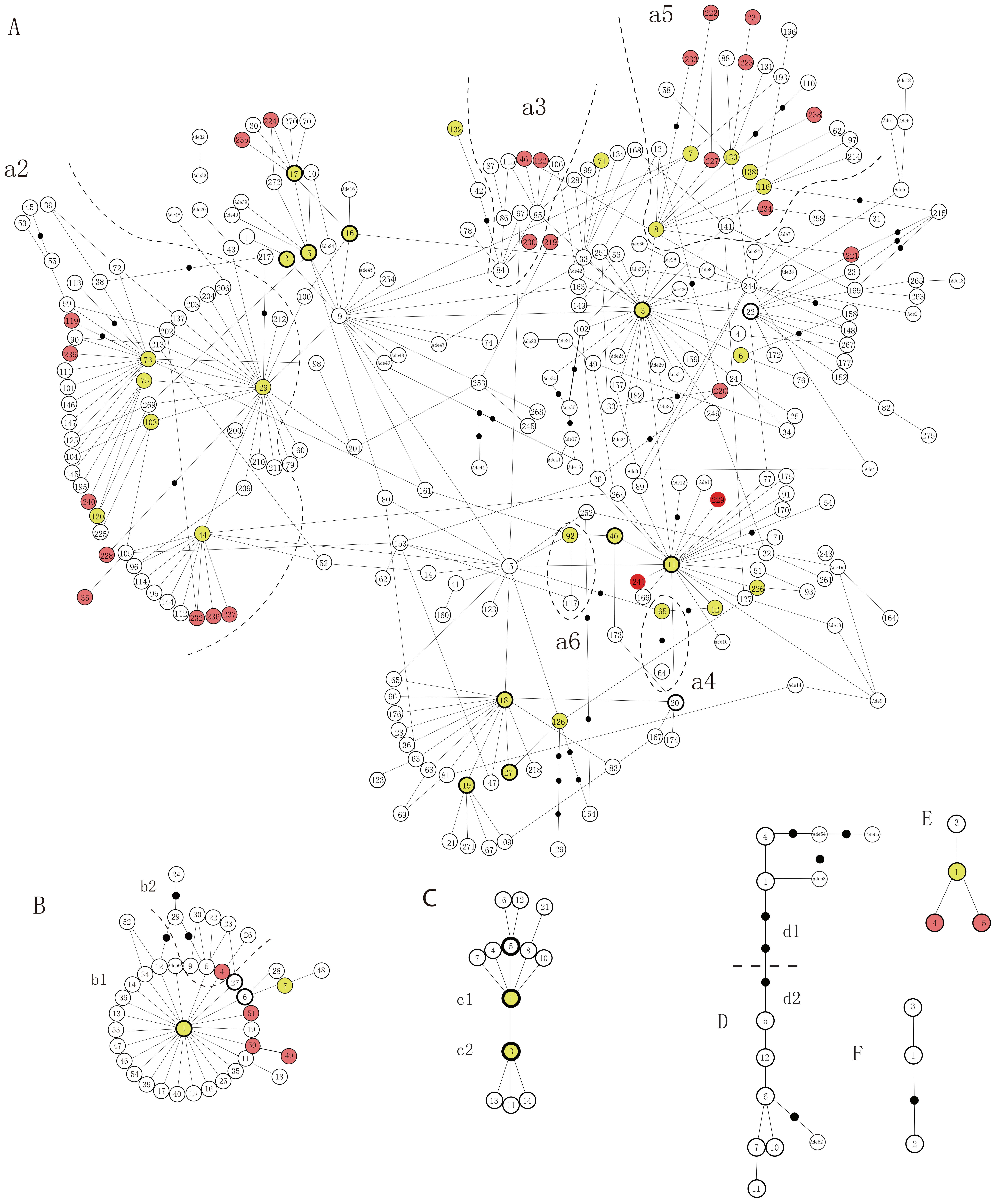

Figure S1b. MS networks showing relationships among haplotypes in Nigeria

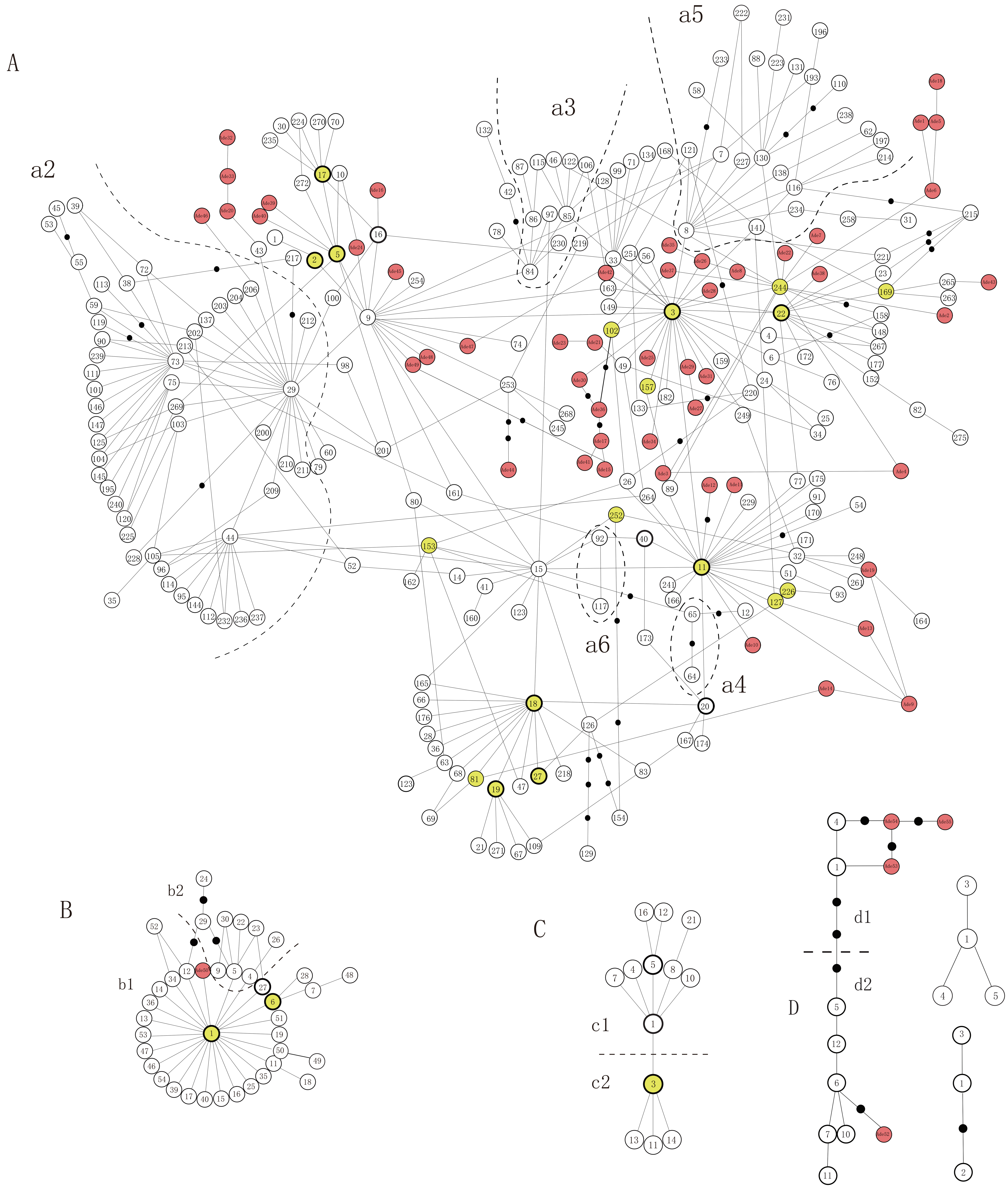

Figure S1c. MS networks showing relationships among haplotypes in sub-Saharan Africa excluding Nigeria

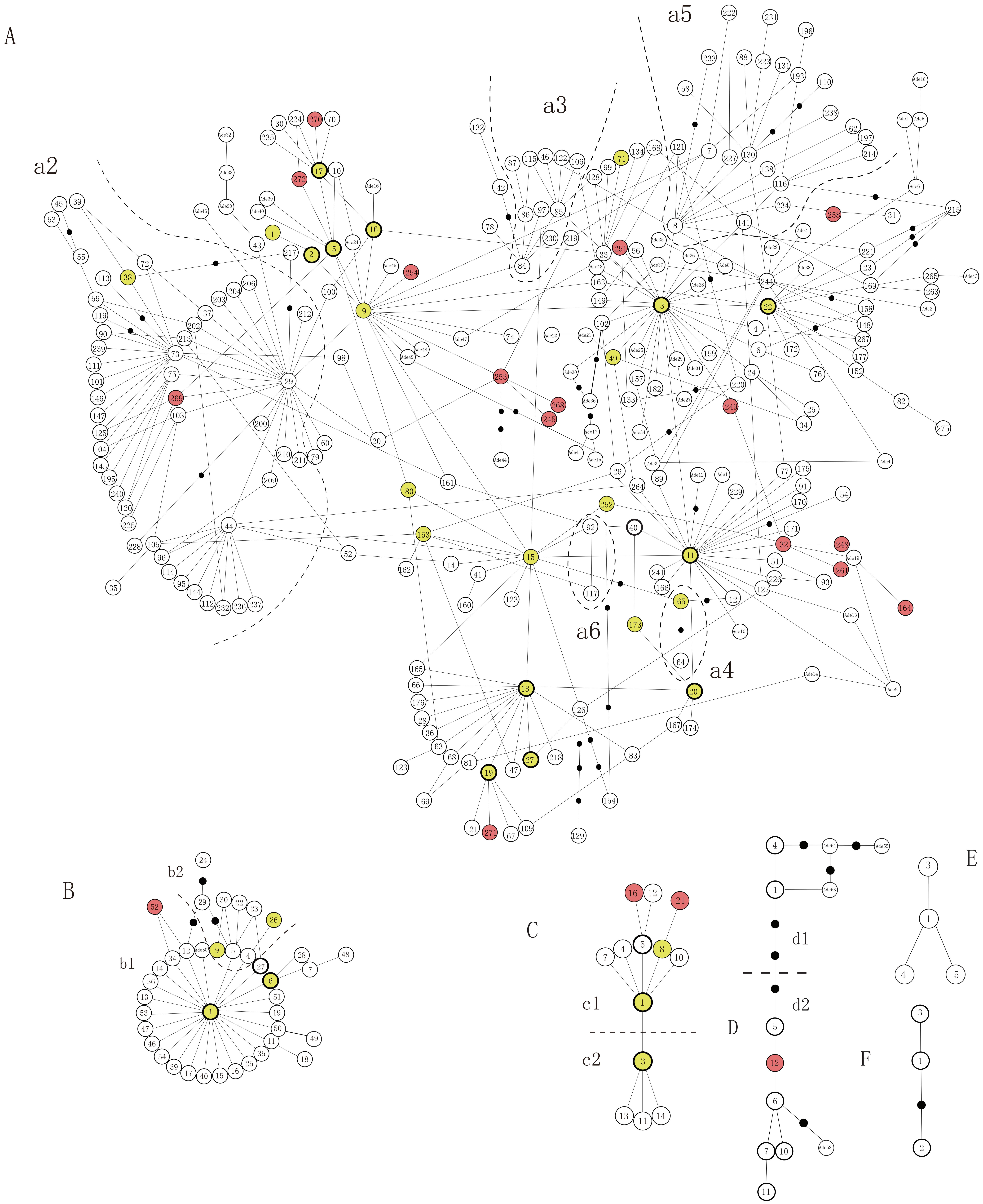

Supplement: Supplementary file 1 [file genes-11-00253-s001.zip › Fig S1.pdf]
