# Supplementary material for: Complete Range of the Universal mtDNA Gene Pool and High Genetic Diversity in the Thai Dog Population
Source: Genes (Basel). 2020 Feb 27;11(3):253. doi: 10.3390/genes11030253 (PMC7140826; doi:10.3390/genes11030253)

Figure S2: The MS network used in Figure 3, showing the distribution of haplotypes in sub-regions of Southern East Asia.

Figure S2a. The MS network used in Figure 3, showing the distribution of haplotypes in North Thailand

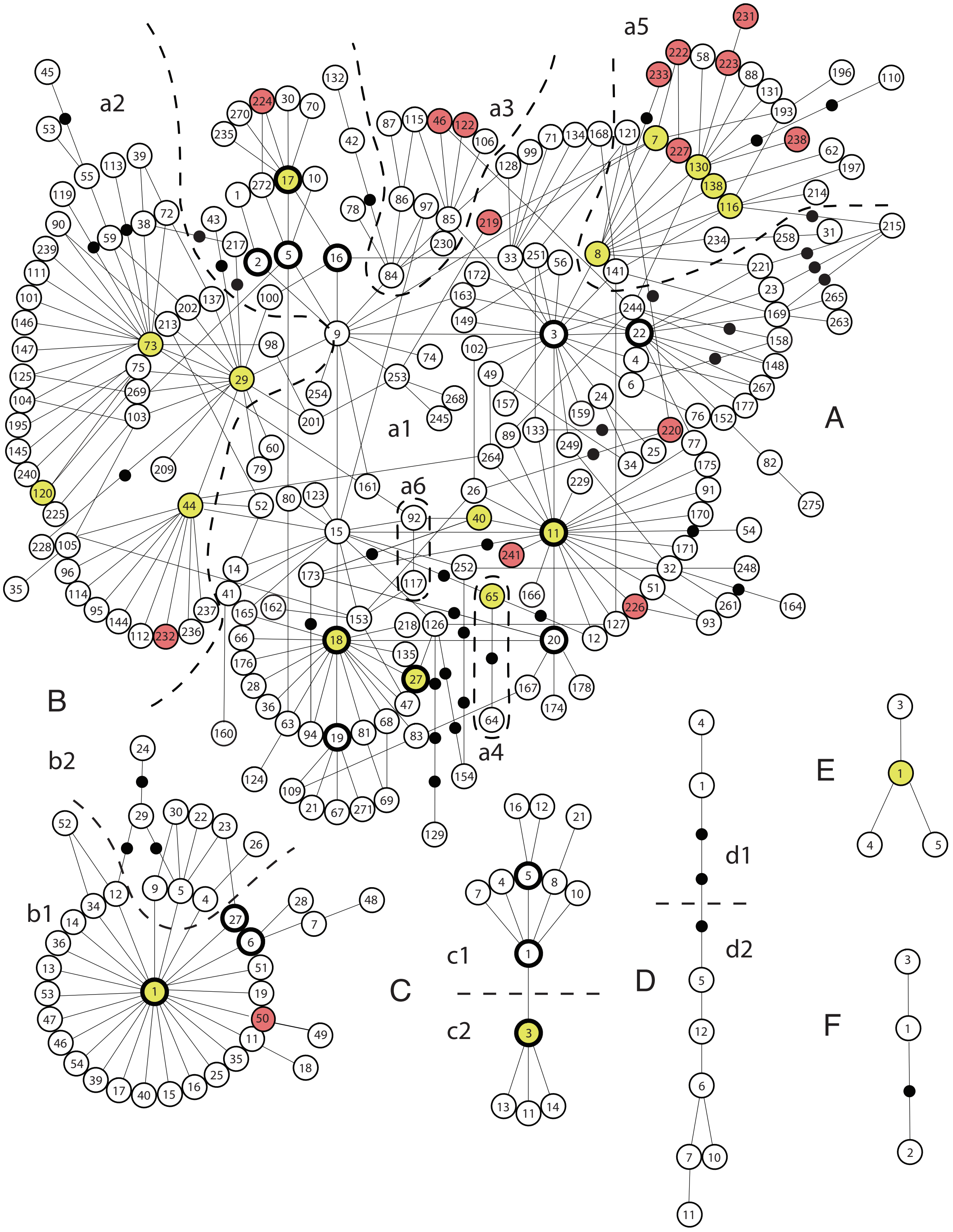

Figure S2b. The MS network used in Figure 3, showing the distribution of haplotypes in Central Thailand

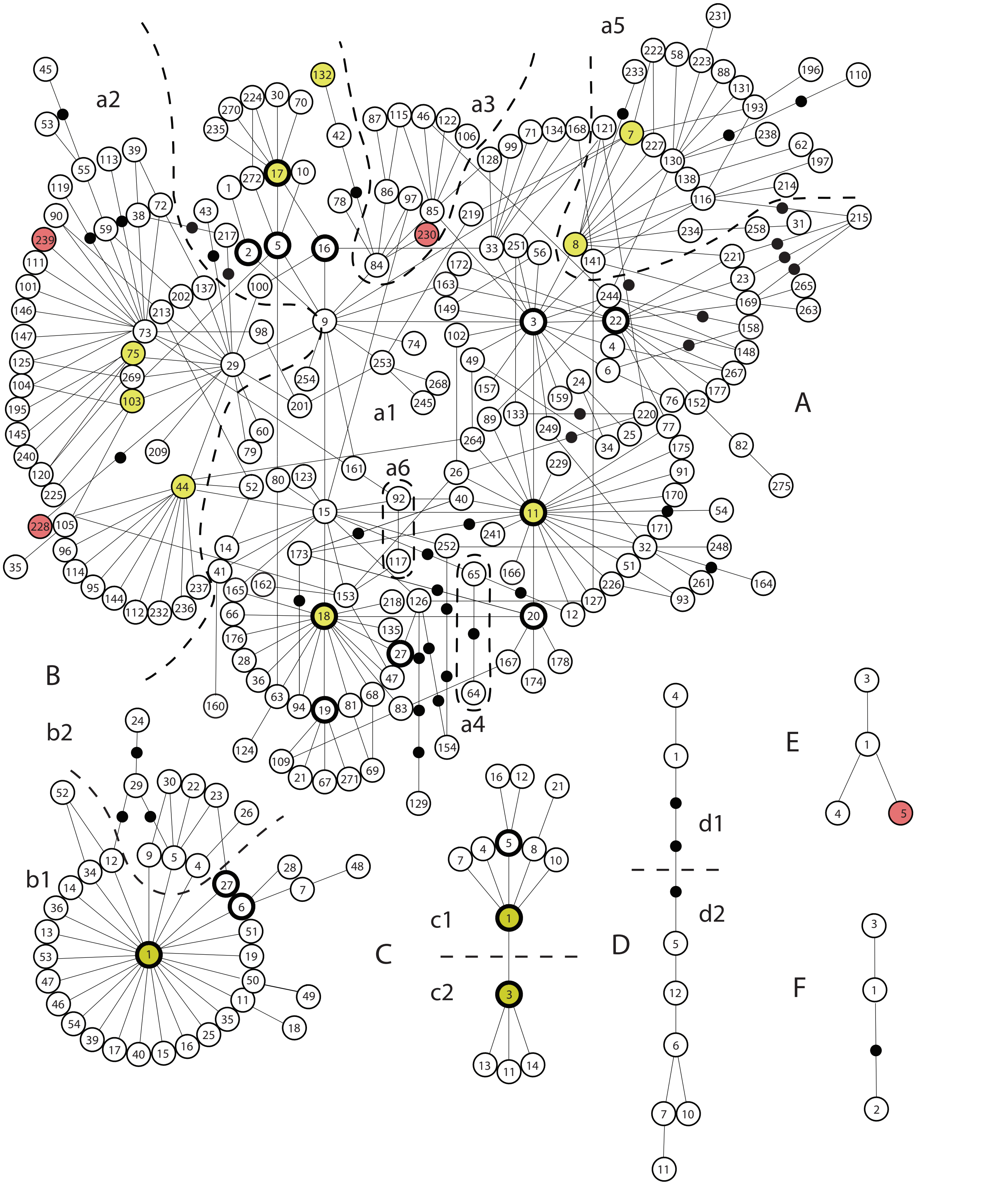

Figure S2c. The MS network used in Figure 3, showing the distribution of haplotypes in Northeast Thailand

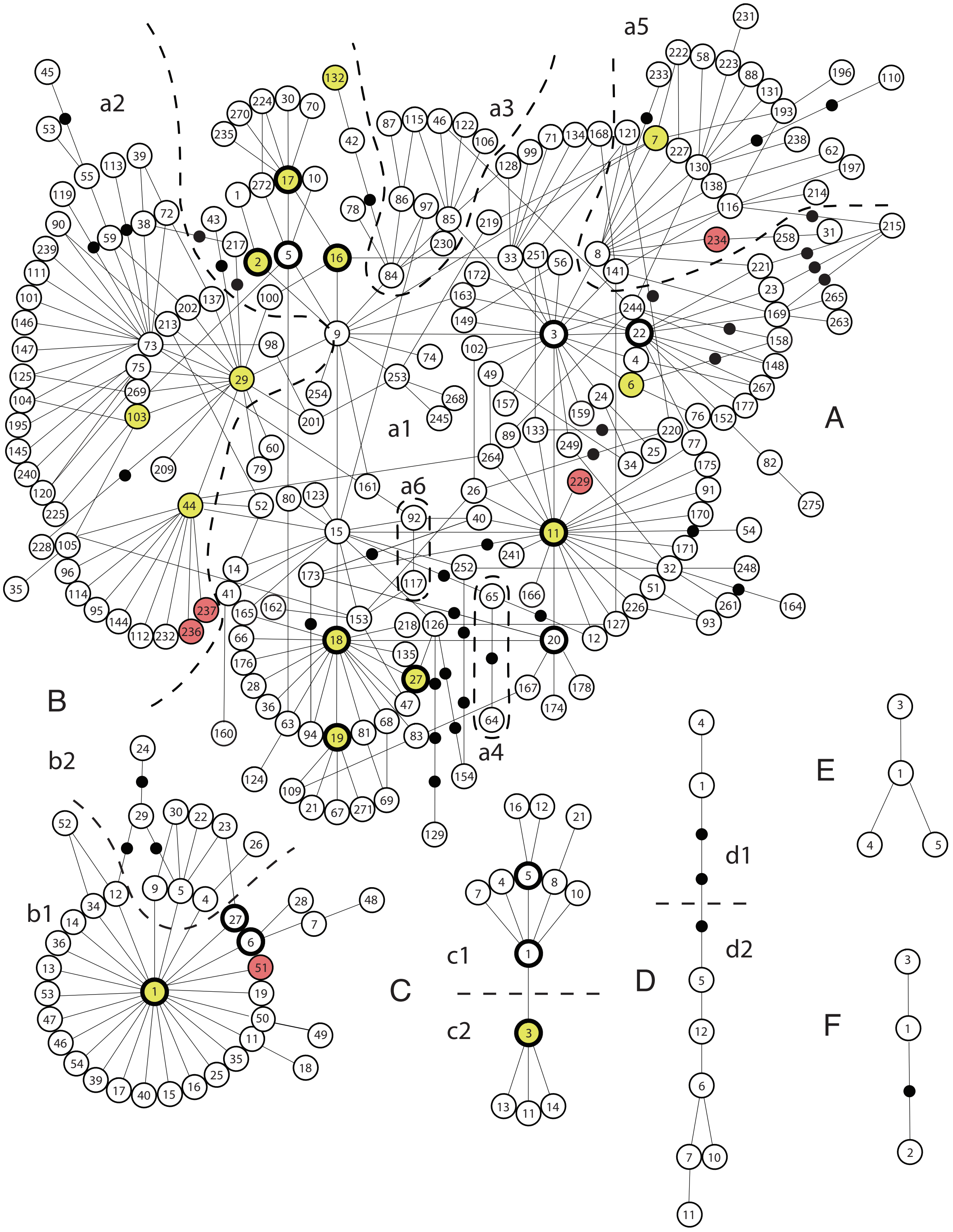

Figure S2d. The MS network used in Figure 3, showing the distribution of haplotypes in South Thailand

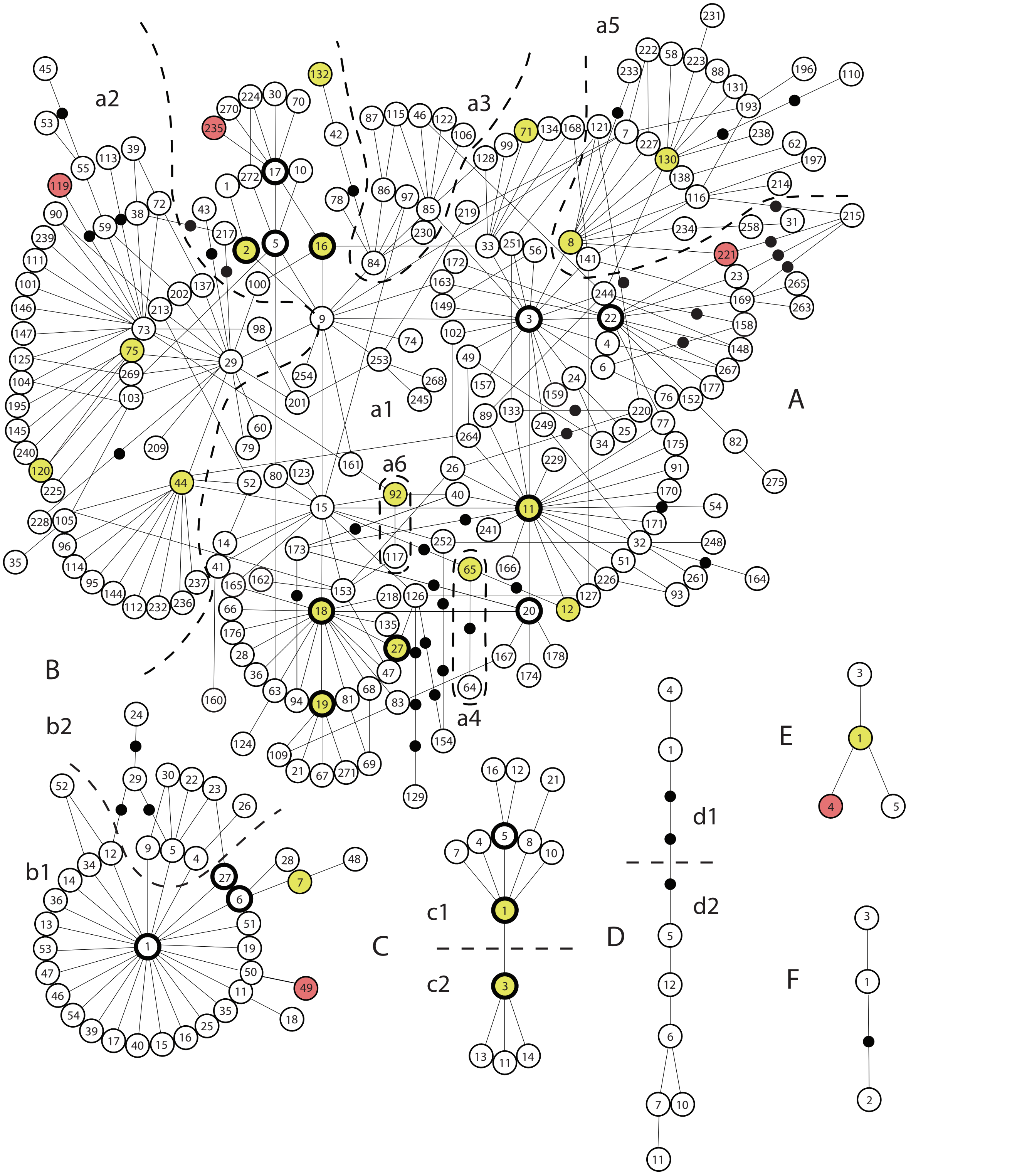

Figure S2e. The MS network used in Figure 3, showing the distribution of haplotypes in Guizhou&Guangxi

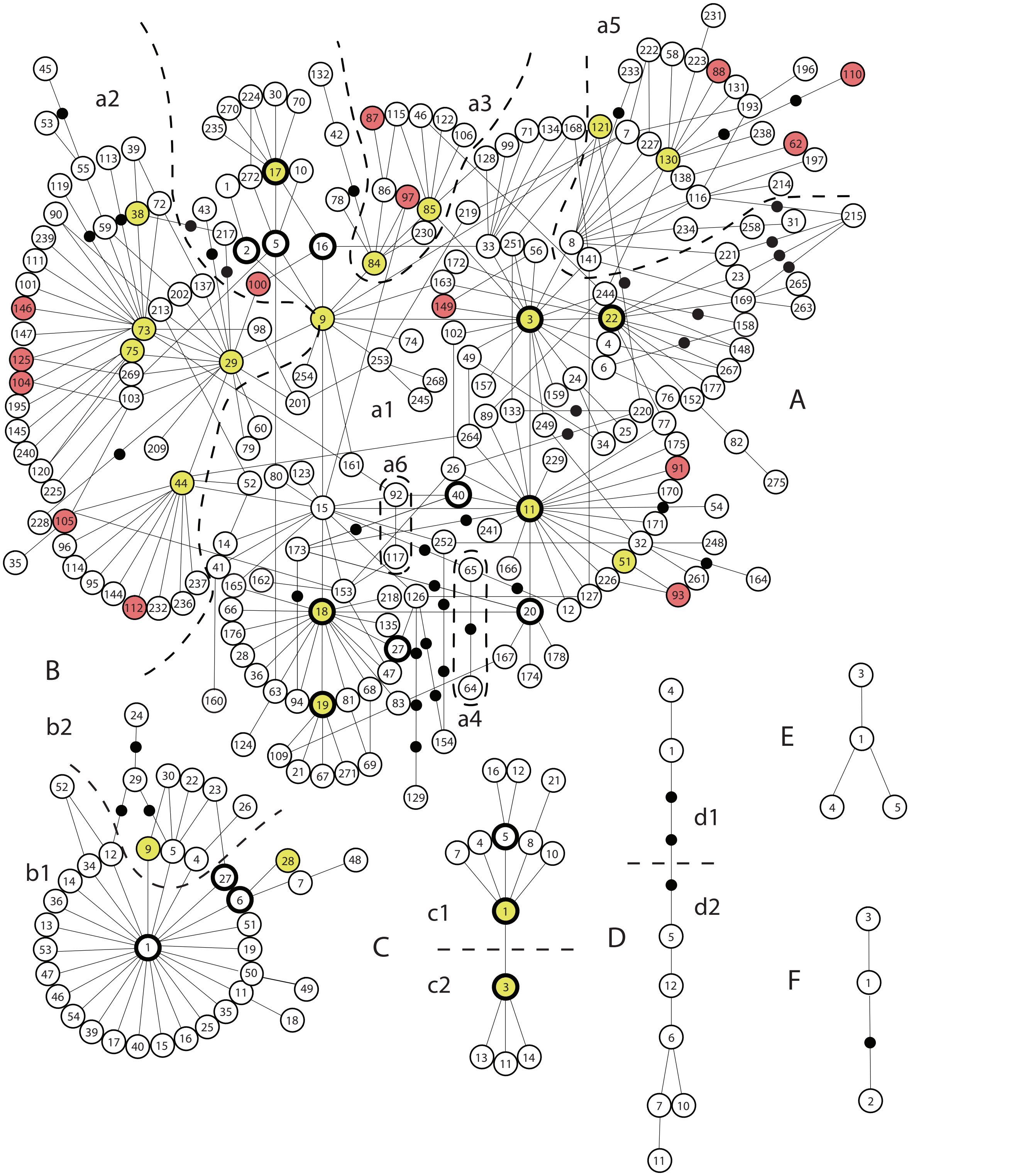

Figure S2f. The MS network used in Figure 3, showing the distribution of haplotypes in Hunan&Jiangxi

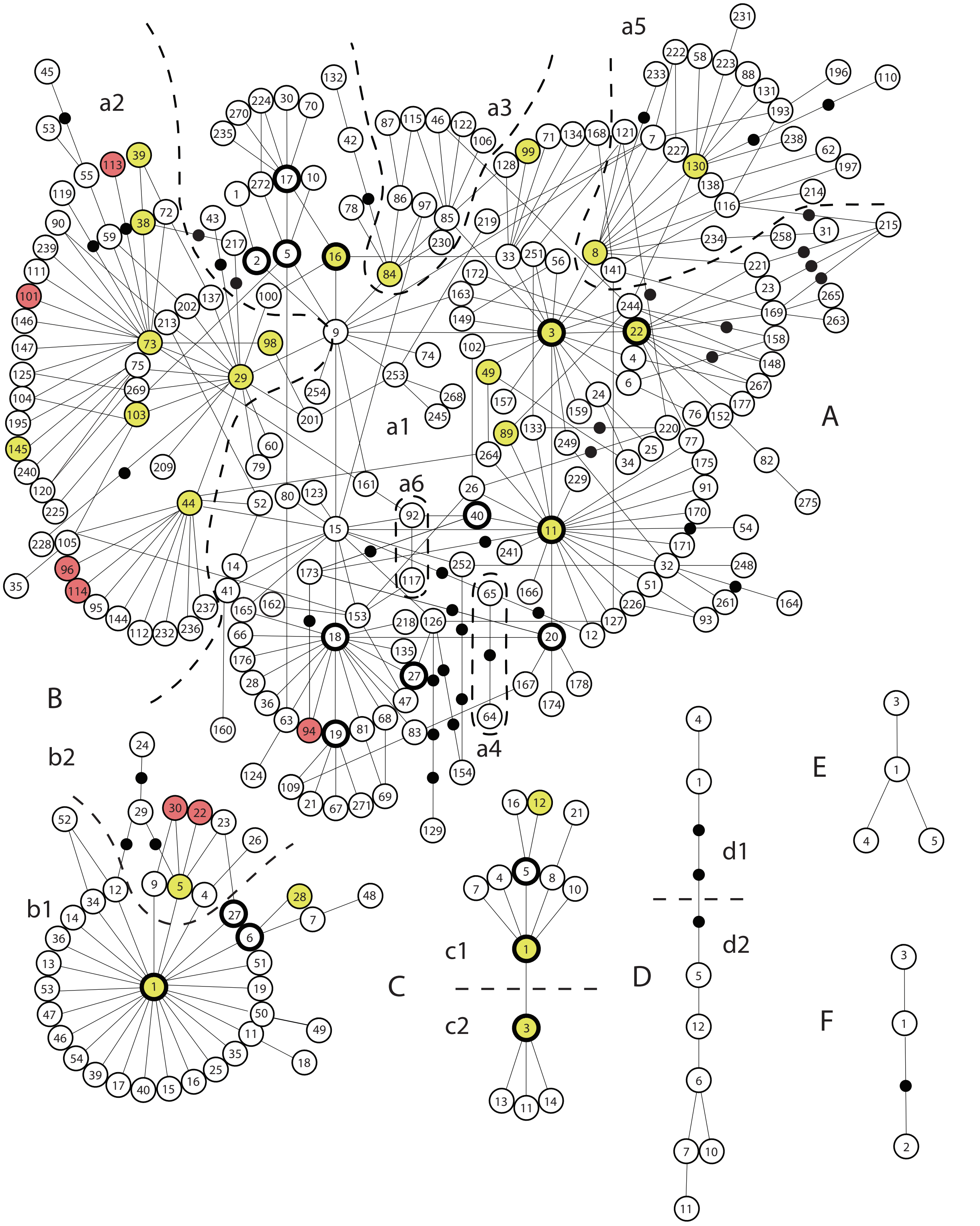

Figure S2g. The MS network used in Figure 3, showing the distribution of haplotypes in Yunnan

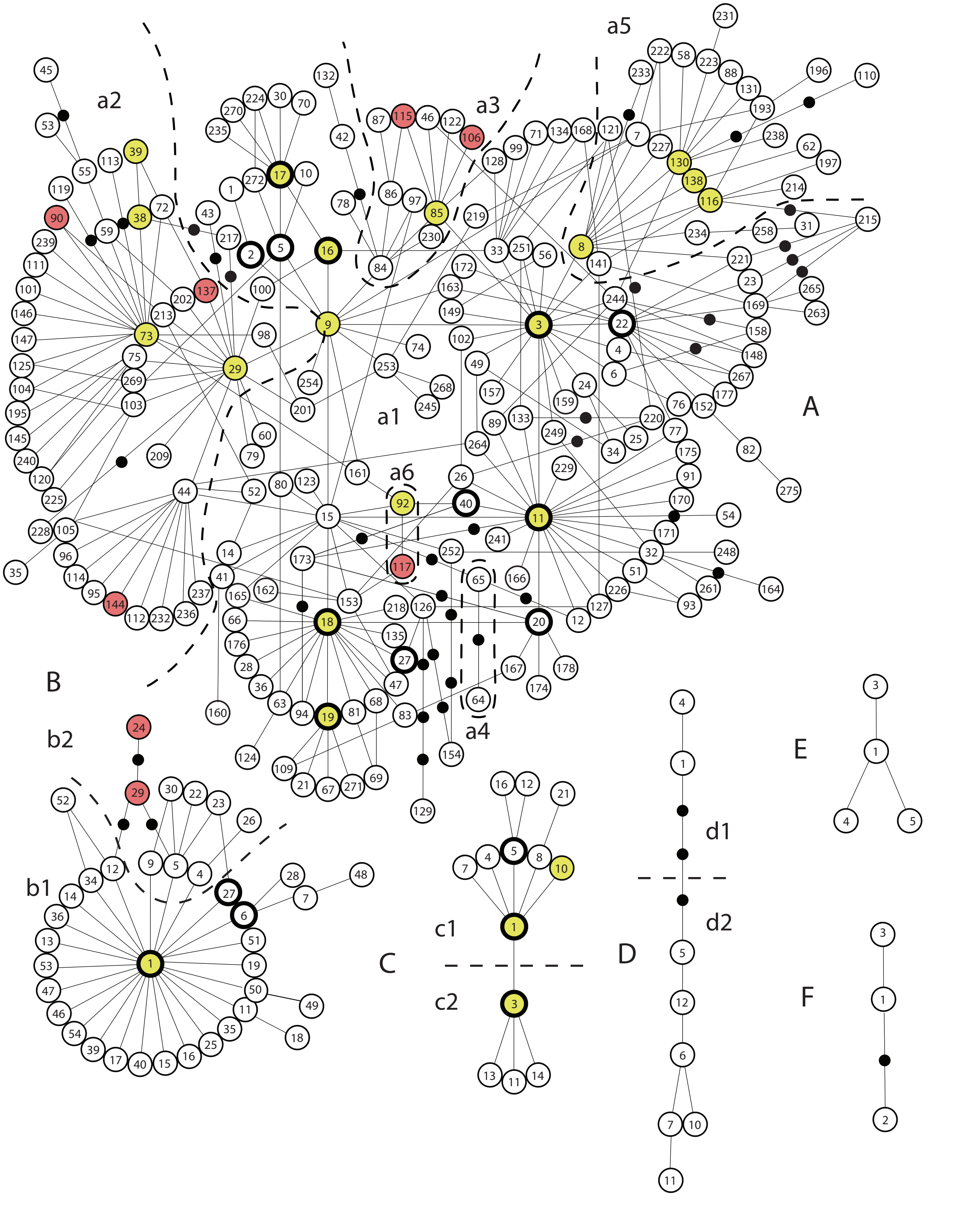

Figure S2h. The MS network used in Figure 3, showing the distribution of haplotypes in Thai ridgeback dogs

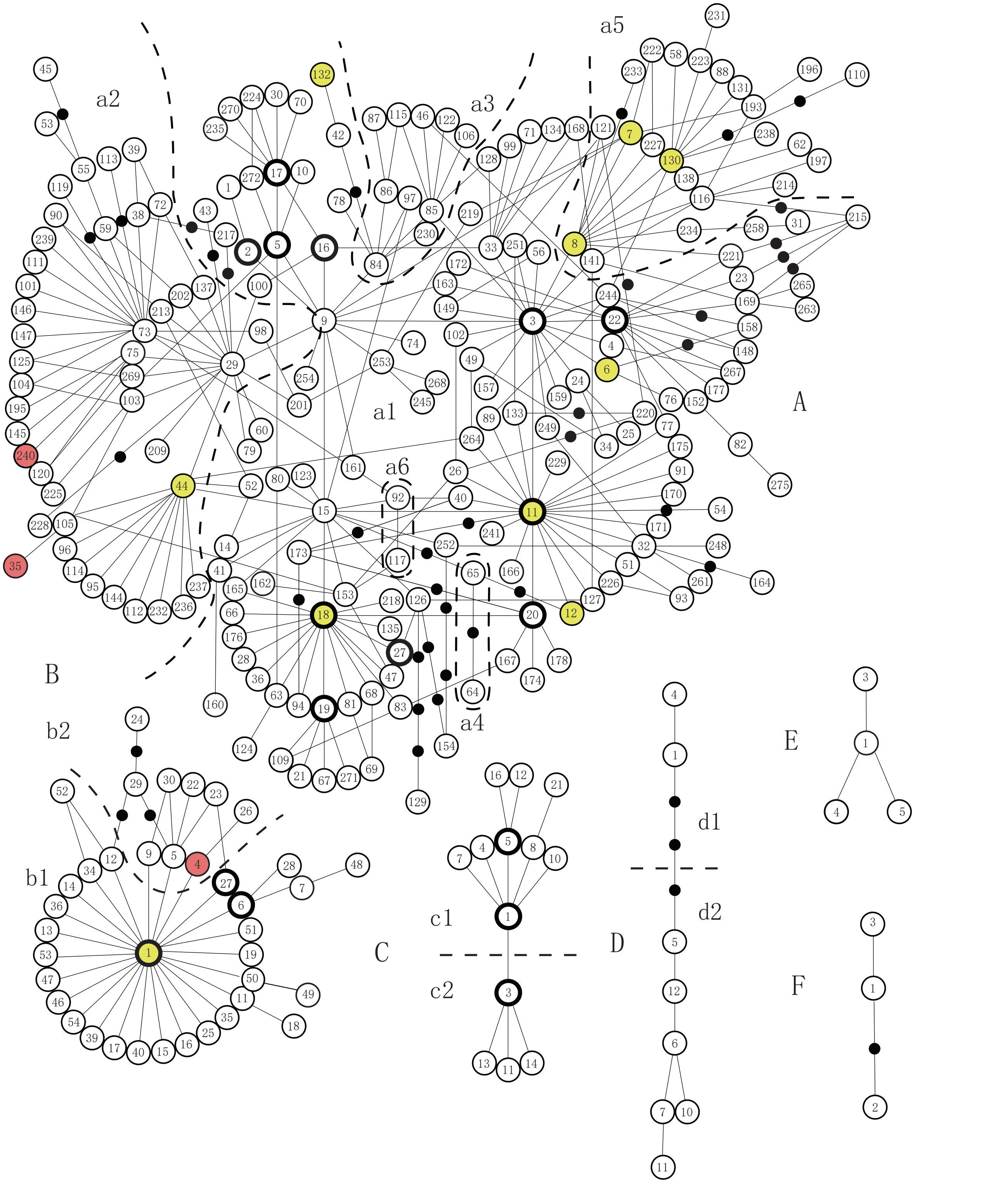

Supplement: Supplementary file 1 [file genes-11-00253-s001.zip › Fig S2.pdf]
