# Supplementary figures and images for: Complete Range of the Universal mtDNA Gene Pool and High Genetic Diversity in the Thai Dog Population
Source: Genes (Basel). 2020 Feb 27;11(3):253. doi: 10.3390/genes11030253 (PMC7140826; doi:10.3390/genes11030253)

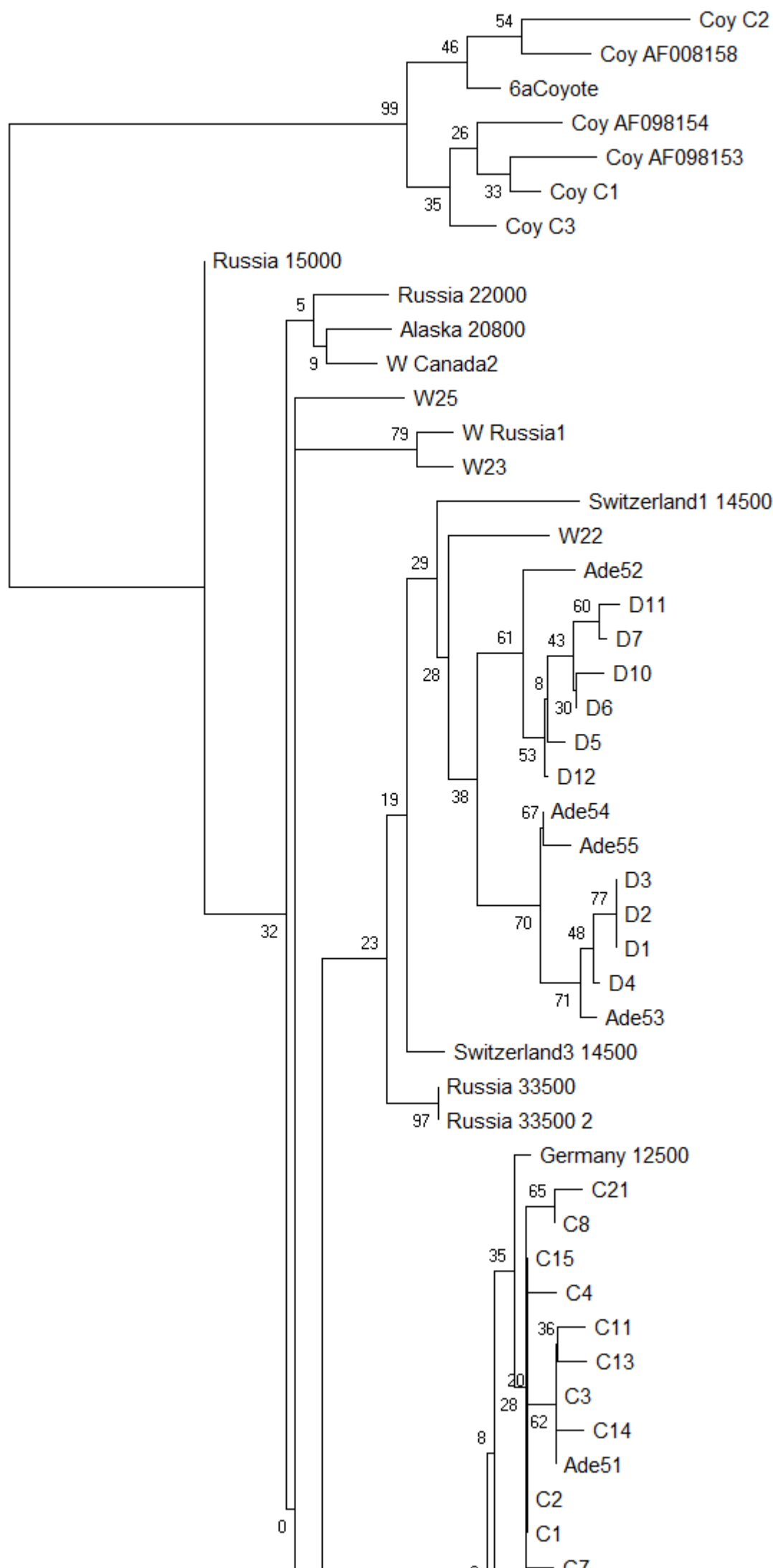

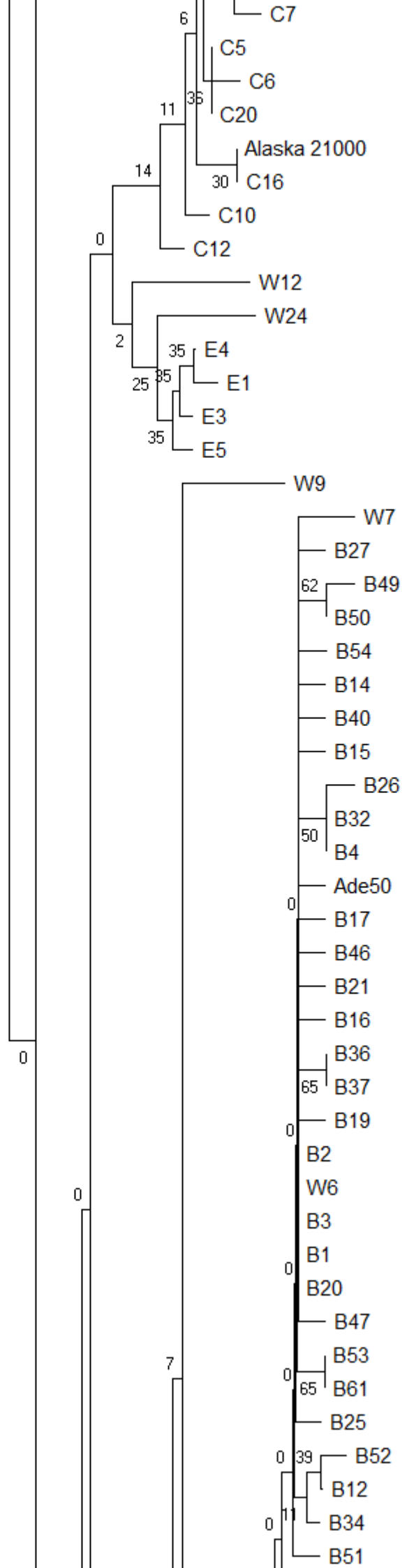

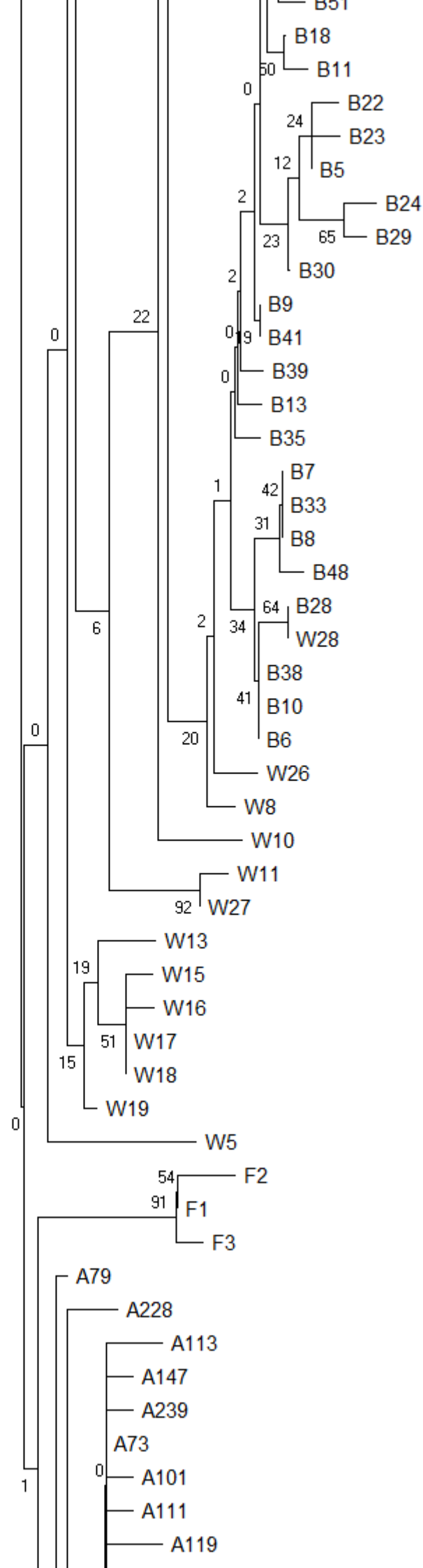

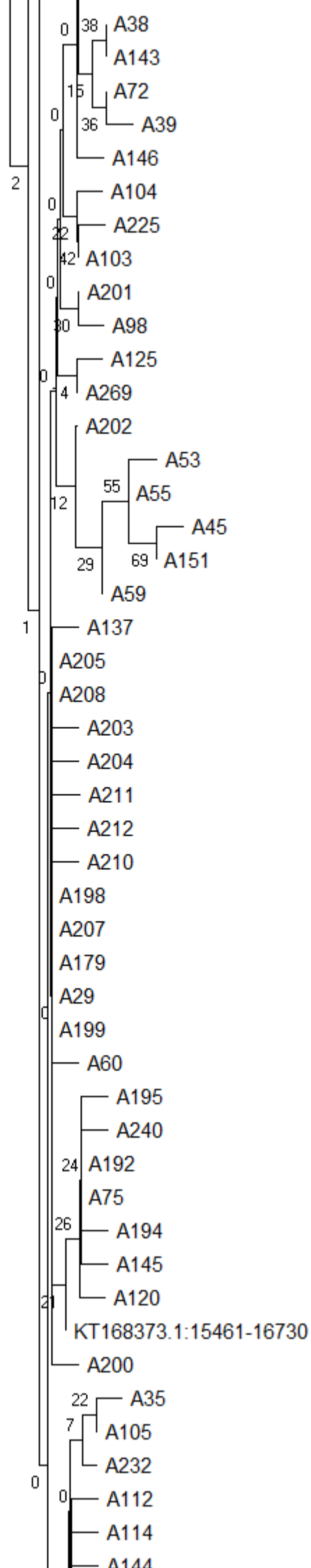

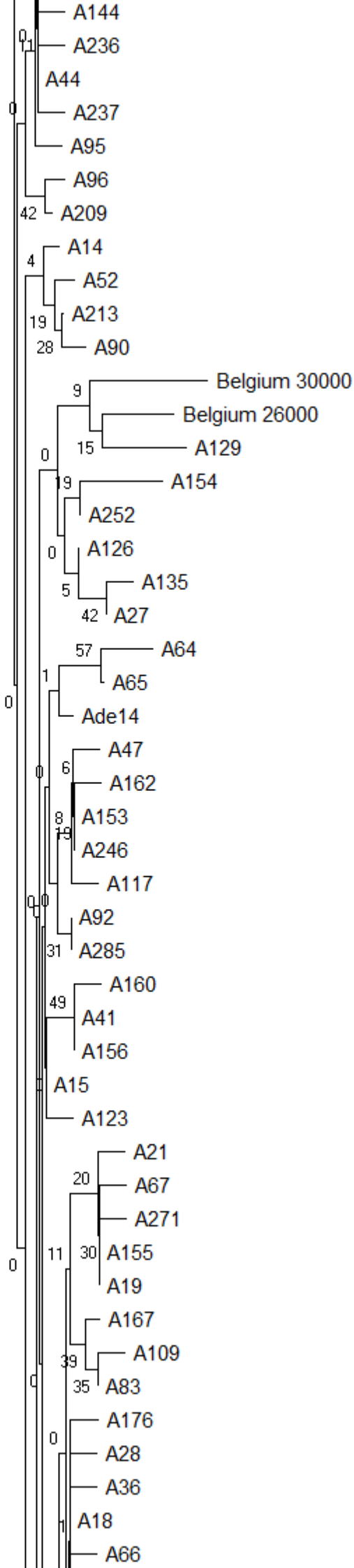

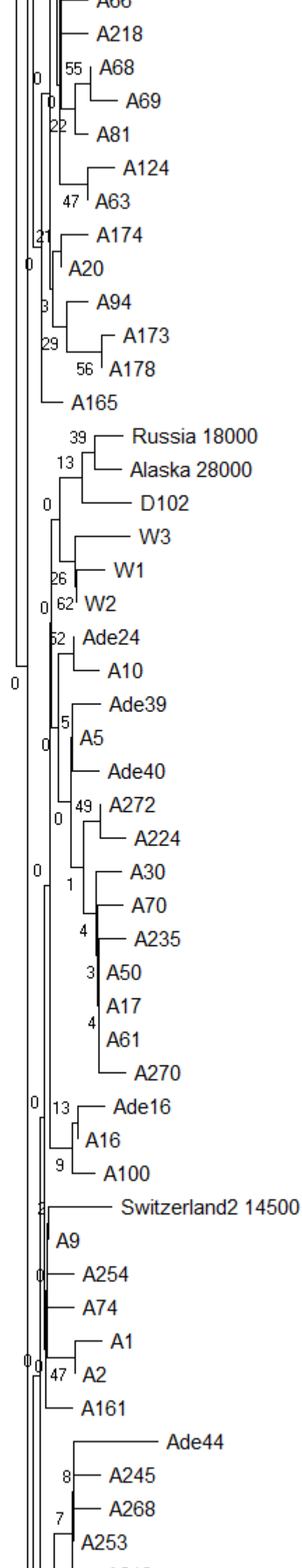

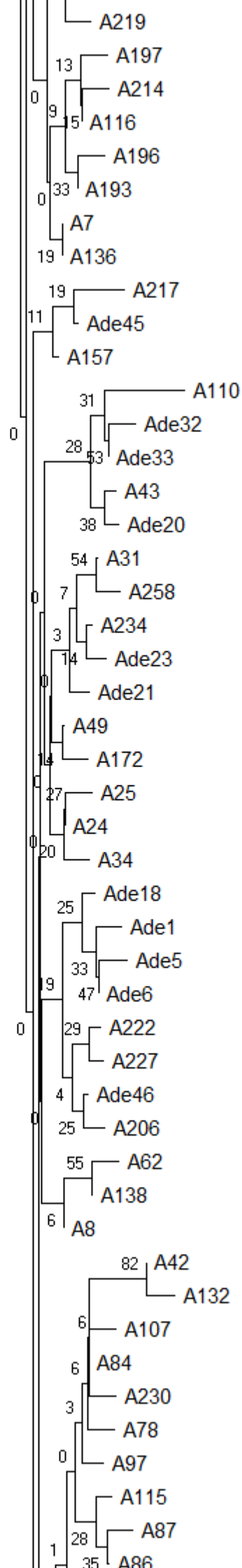

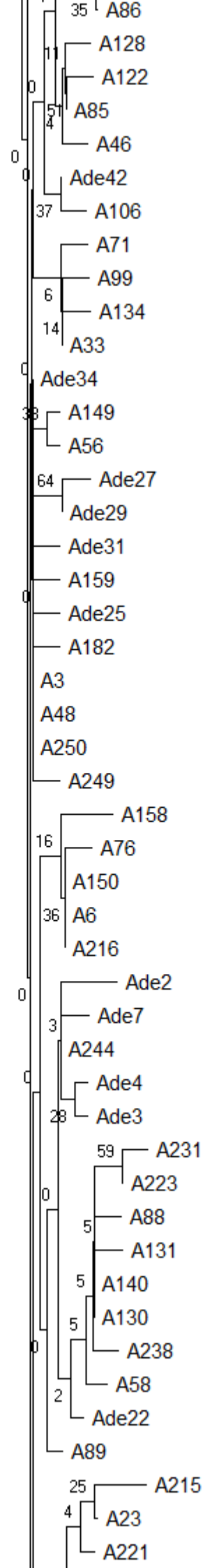

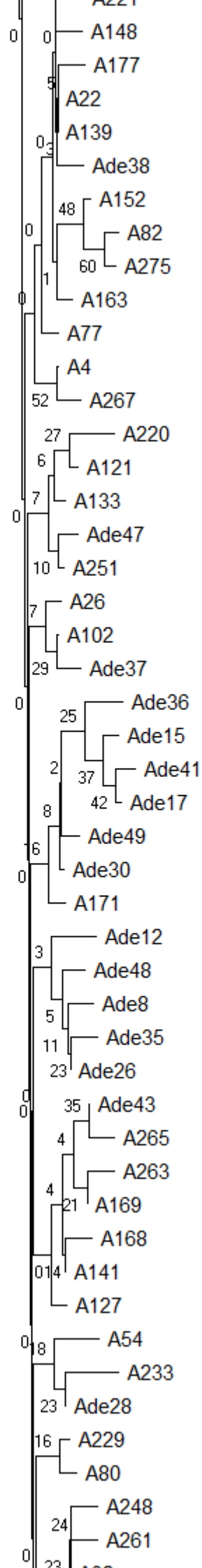

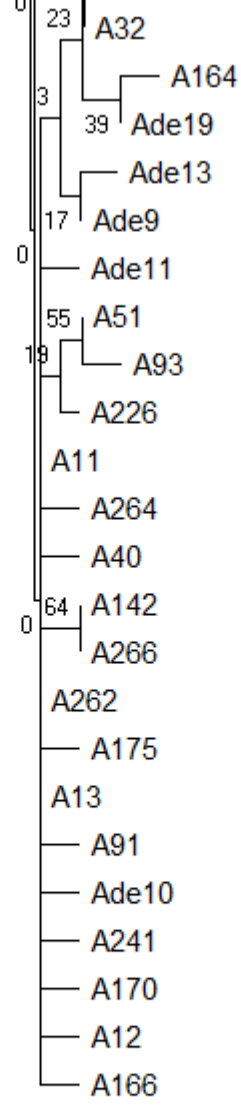

0.010

Supplement: Supplementary file 1 [file genes-11-00253-s001.zip › Fig S3.pdf]
